# Living birth following preimplantation genetic testing for monogenic disorders to prevent low-level germline mosaicism related Nicolaides–Baraitser syndrome

**DOI:** 10.3389/fgene.2022.989041

**Published:** 2022-09-09

**Authors:** Jiexue Pan, Jie Li, Songchang Chen, Chenming Xu, Hefeng Huang, Li Jin

**Affiliations:** ^1^ Obstetrics and Gynecology Hospital, Institute of Reproduction and Development, Fudan University, Shanghai, China; ^2^ Shanghai Ji Ai Genetics and IVF Institute, Obstetrics and Gynecology Hospital, Fudan University, Shanghai, China; ^3^ Shanghai Key Laboratory of Embryo Original Diseases, Shanghai, China; ^4^ Research Units of Embryo Original Diseases, Chinese Academy of Medical Sciences, Shanghai, China

**Keywords:** sperm mosaicism, prenatal genetic testing for monogenic disorders, recurrent monogenic disease, SMARCA2, Nicolaides–Baraitser syndrome

## Abstract

**Objective:** Paternal sperm mosaicism has few consequences for fathers for mutations being restricted to sperm. However, it could potentially underlie severe sporadic disease in their offspring. Here, we present a live birth of a female infant from a father with low-level sperm DNA mosaicism achieved *via* preimplantation genetic testing for monogenic disorders (PGT-M).

**Methods:** A couple with the father carrying sperm DNA mosaicism received standard *in vitro* fertilization treatment, with intracytoplasmic sperm injection, embryo biopsy, polymerase chain reaction, and DNA analysis. Only one unaffected embryo was transferred to the uterine cavity. Amniocentesis was performed at the 16th week of gestation by copy-number variation-sequencing, karyotyping, and Sanger sequencing.

**Results:** Eight surviving embryos were biopsied during the blastocyst stage. Karyomapping and Sanger sequencing were applied to detect the euploidy and paternal mutation. After performing PGT-M, followed by successful pregnancy, the prenatal genetic diagnoses revealed that the fetus was unaffected, and one healthy girl was born.

**Conclusion:** This is the first reported live birth with unaffected children achieved *via* PGT for a low-level germline mosaicism father. It not only opens the possibility of preventing the recurrent monogenic disease of children among gonadal mosaicism families but also alerts clinicians to consider gonadal mosaicism as the source of DMNs.

## Introduction

Mosaicism, defined as a condition where two or more genomes in an individual derived from a single zygote, could be classified into gonadal mosaicism, somatic mosaicism, and gonosomal mosaicism (the co-existence of gonadal and somatic mosaicism) (Biesecker and Spinner, 2013). Sperm mosaicism specifically refers to a man’s sperm cells carrying genetic variants that are not constitutively present in somatic cells. Any type of genetic variant can occur in the sperm, including single-nucleotide variations (SNVs), nonrecurrent copy-number variations (CNVs), small insertions and deletions (INDELS), and copy-number-neutral structural variations (Breuss et al., 2021). As the genetic information in the father’s sperm can be transmitted to the next generation, sperm mosaicism could potentially be present in any cases reported as *de-novo* mutations (DNMs) in the child and cause a large proportion of severe sporadic diseases in childhood (Telenius et al., 1995; Zhang et al., 2019; Breuss et al., 2020; Lin et al., 2020). Depending on different properties, the recurrence rate of the DNMs could range from 0.011% to 28.5% (Jonsson et al., 2018).

Nicolaides–Baraitser syndrome (NCBRS) (OMIM 601358), caused by a pathogenic mutation in the SMARCA2 gene, is an autosomal dominant disease going along with intellectual delay, facial coarsening, short stature, seizures, and prominent interphalangeal joints (Sousa et al., 2014). More than 80 individuals with NCBRS caused by a mutation in the SMARCA2 gene have been reported worldwide, and 58 were caused by DNMs (Zhang et al., 2022).

We have reported a case for being the first sperm mosaicism case of SMARCA2-related disease (Pan et al., 2021). The mosaicism seemed to be restricted to the paternal gonad because of undetectable pathogenic mutation in other somatic cells, including blood or buccal cells. The level of mosaicism for the pathogenic allele is relatively low (2.88%), but two successive children with the same pathogenic variant were affected.

Herein, we prevented the transmission of pathogenic mutation and legal interruption of pregnancy in a family with the help of preimplantation genetic testing for monogenic disorders (PGT-M) and prenatal genetic diagnosis (PND).

PGT is an alternative method for conventional prenatal diagnosis. PGT refers to the genetic analysis of an embryo, involving multiple assisted reproductive procedures, including superovulation, intracytoplasmic sperm injection (ICSI), *in vitro* embryo culture, blastomere biopsy, vitrification, and genetic testing. It has been sophisticatedly used for preventing abnormal pregnancy in hereditary monogenic disorders, aneuploidy, and chromosomal rearrangements (Handyside et al., 1990; Shi et al., 2021). Recently, a cohort study assessed and demonstrated the transmission of gonadal mosaic mutations to preimplantation blastocysts with PGT (Breuss et al., 2022). Though it has been reported that a somatic-gonadal mosaicism parent had unaffected offspring through PGT (Rechitsky et al., 2011), there is no successful report of preventing monogenic disease of offspring caused by paternal mosaicism restricted to gonad in literature. Here, we first report a successful pregnancy with a genetically normal child achieved *via* PGT for a father carrying mutant disease-causing SMARCA2 variant restricted to his sperm.

## Materials and methods

### Clinical presentation

**FIGURE 1:**
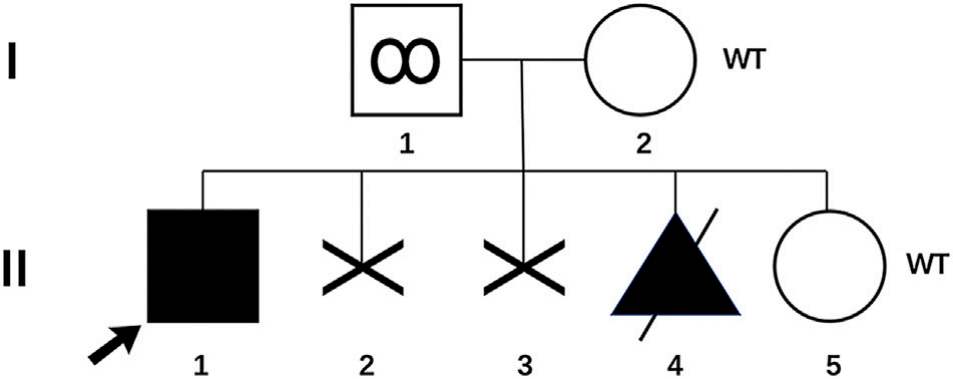
Pedigree of the family with genotype results for *p*. Gln185Glu variant. (I-1) Father with *p*. Gln185Glu variant in his sperm; (I-2) mother of the proband; (II-1) proband with *p*. Gln185Glu variant; (II-2 and 3) two aborted fetuses, unknown genotypes; (II-4) aborted fetus with *p*. Gln185Glu variant; (II-5) infant born through PGT-M. WT, wild type.

We report a family with a gravida 4/para 1/abortion 3 mother planning to have their second child (Figure 1). The parents were healthy and denied consanguinity. In 2011, their first boy was born by full-term normal delivery. No pregnancy or birth complications were recalled. The boy presented with intellectual delay, autistic characteristics, and intractable epilepsy after anti-epileptic treatment since the age of 2. The diagnosis of the boy was unclear until genetic testing in 2018, and the mother suffered two unintended pregnancies and induced abortions during this period. The genetic testing revealed a heterozygous pathogenic mutation in the affected boy (SMARCA2 (NM_003070.5: c.553C>G, *p*. Gln185Glu)). Since a familial trio clinical exome sequencing showed that neither of the parents carried the mutation, the possibility of *de novo* variation was considered. In 2019, the couple planned to have another child and received pre-pregnancy genetic counseling. Considering the low risk for the recurrence of DNMs, local doctors suggested them a natural pregnancy. In 2020, the wife got pregnant, and a prenatal genetic diagnosis was performed at the 18th week of gestation. Unfortunately, the same pathogenic variation was detected in amniocytes, and the woman underwent induced abortion again. The mosaicism of sperm carrying pathogenic allele was reported and verified as performed previously (Pan et al., 2021). The mosaicism is also presented in Figure 1.

### DNA extraction and Sanger sequencing

Genomic DNA was extracted using DNeasy Blood and Tissue kits according to the protocol (Qiagen, Germany). In brief, the genomic regions which contain the missense SMARCA2 variant were targeted with the reference sequence (NG_032162.2, transcript: NM_003070.5) and then amplified specifically based on the primers (forward: 5-CCA​ACA​GAG​GTC​CCT​CAC​CT-3, reverse: 5-GCC​TCG​GGC​CAG​CAT​TTT​AT-3). The PCR conditions were as previously described (Pan et al., 2021).

### Haplotype construction

SNP flanking 2 Mb of the pathogenic sites were selected for linkage analysis. We performed multiplex PCR technology for the genomic DNA of the proband. We analysed the genotype sites to screen the informative SNPs loci where the proband was heterozygous and the mother was homozygous.

### Prenatal genetic testing for monogenic disorders and prenatal genetic diagnosis

In brief, oocytes were retrieved from the woman *via* vaginal ultrasound-guided aspiration, and metaphase II (MII) oocytes were fertilized *via* ICSI with her husband’s sperm and then cultured for 5–6 days to the blastocyst stage. Three to five trophoblast ectoderm cells were obtained as samples by biopsy and transferred to PCR tubes containing phosphate buffer saline immediately for whole genome amplification (WGA) by multiple displacement amplification (MDA) technology. WGA was performed using the Repli-g Single Cell Kit (Qiagen, Hilden, Germany). The WGA products of the embryo biopsies and the gDNA of parents and proband were used for karyomapping. Using the single-nucleotide polymorphism (SNP)-array technology, karyomapping was performed according to the Infinium Karyomapping assay protocol (Illumina, San Diego, CA, United States). The HumanKaryomap-12 (v1.0) DNA analysis kit, iScan System, and BlueFuse Multisoftware were involved in the scanning and reporting of the analysis. SNP linkage and chromosomal aneuploidy analysis were conducted based on SNP-array data. Sanger sequencing was also performed as previously described (Pan et al., 2021). Embryos diagnosed as unaffected were selected for transfer. The presence of a gestational sac by ultrasound 28 days after embryo transfer was defined as clinical pregnancy, and further routine prenatal care was taken in the obstetrics department of our hospital.

For PND, amniocentesis was performed at the 16th gestational week. Amniotic fluid cells were used for chromosomal karyotyping, genomic CNV sequencing, and Sanger sequencing for the verification of chromosomal normality and the absence of pathogenic SMARCA2 variants as previously described (Shi et al., 2021).

## Results

**FIGURE 2:**
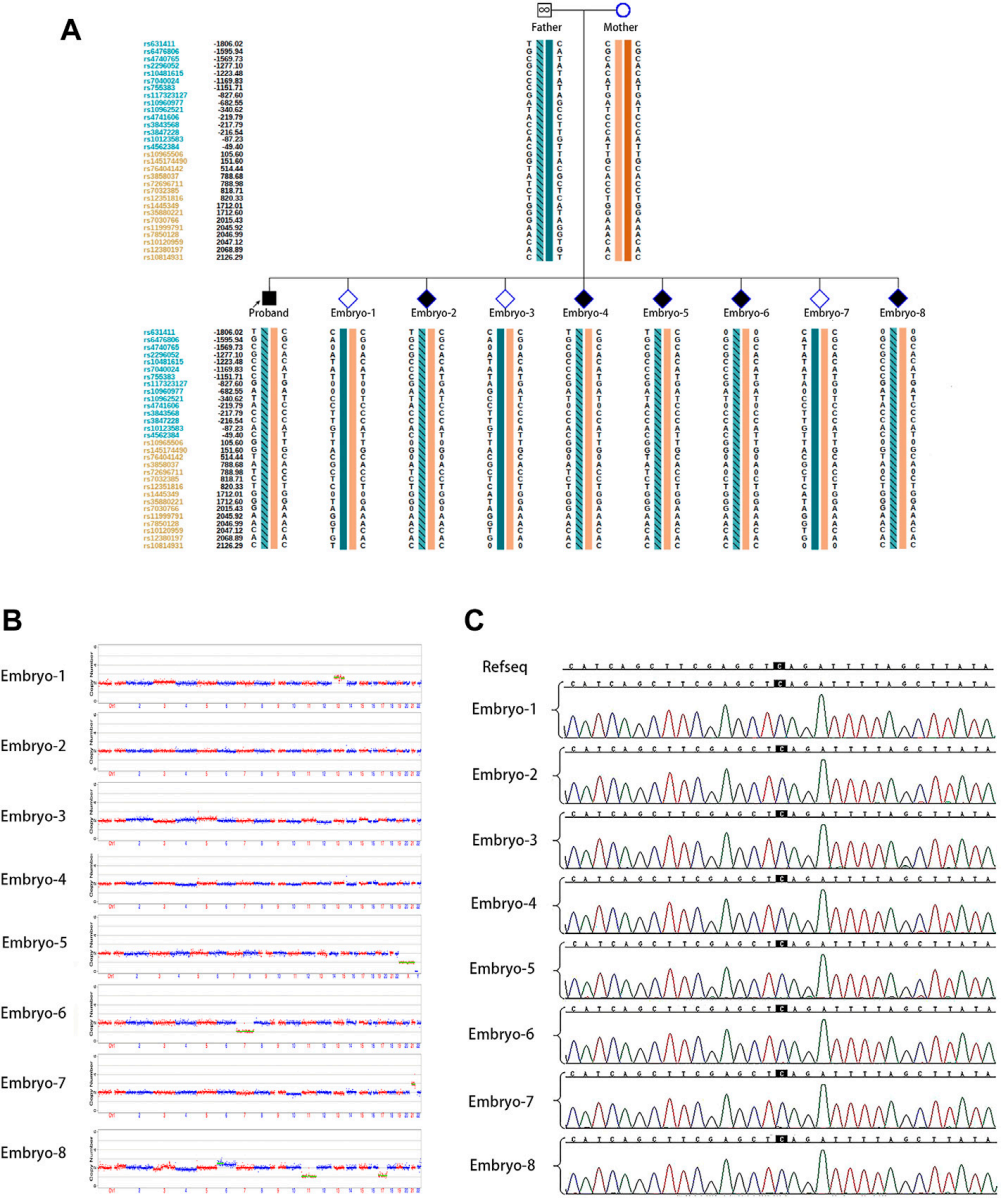
PGT for the SMARCA2 variant. **(A)** Results of haplotype linkage analysis in SMARCA2 gene in parents. PGT haplotype analysis in embryos 1 to 8. Orange band means chromosome from mother, cyan band without slashes means normal chromosome from father, and cyan band with slashes means chromosome from father. Embryos 1, 3, and 7 showed the absence of disease-causing variants.; **(B)** PGS revealed that embryos 2, 3, and 4 were euploidy; **(C)** Sanger sequencing of DNA from all eight embryos showed the absence of SMARCA2 variants at c 533.

**FIGURE 3:**
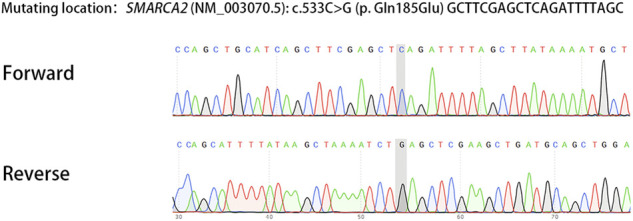
Sanger sequencing of DNA from amniotic fluid cells showed the absence of SMARCA2 mutation.

The couple was referred to the reproductive genetics clinic of Obstetrics and Gynecology Hospital for preconception counselling. Comprehensive counselling was provided by an experienced gynecologist and a geneticist. Considering the medical history of two successive affected children with the same pathogenic variant and low level of mosaicism in sperm, PGT-M or natural conception plus prenatal diagnosis was regarded as an alternative option. The couple eagerly opted for PGT-M for unaffected children. Following the gonadotrophin-releasing hormone (GnRH) antagonist protocol, 23 oocytes were retrieved with the guidance of ultrasound, and 20 MII oocytes were fertilized *via* ICSI. Eight surviving embryos reached the blastocyst stage. The detection of mutated alleles and chromosomal abnormalities were performed simultaneously using karyomapping. In addition, Sanger sequencing was also performed to verify the pathogenic locus of the proband. Three embryos were euploid (Figures 2A,B). Fortunately, one euploid embryo was detected free of the heterozygous paternal mutation among the three euploid embryos (Figure 2A). Finally, the euploid embryo without the pathogenic mutation (Figures 2B,C) was transferred into the uterus, which resulted in an intrauterine singleton pregnancy. Amniocentesis was performed at 16 weeks of pregnancy, and DNA from amniotic fluid cells were detected with no SMARCA2 mutation *via* Sanger sequencing. Meanwhile, chromosomal karyotyping and CNV-sequencing showed normal results (Figure 3). The baby was born at 39 weeks gestation and was healthy.

## Discussion

Here, we present the first case of a successful pregnancy for a father with mosaicism restricted to gonad conceived *via* PGT-M. This father having a normal phenotype carries the mutant SMARCA2 gene in his sperm and possibly transmits the pathogenic variation to his child.

Gonadal mosaicism has been reported in a variety of dominant (Monies et al., 2019) or X-linked conditions (Zhong et al., 2021) and should be considered in all cases of apparent *de-novo* variation. Sperm mosaic can cause a wide variety of diseases as many types of genetic perturbation can occur in the sperm (Bell et al., 2020; Breuss et al., 2020; Lin et al., 2020; Li et al., 2021). It means that sperm mosaic without a visible phenotype is probably underestimated in cases of DNMs when only one fetus or child is affected. The majority of DNMs have a slim recurrence risk, but they could have a substantially higher risk when having origins in sperm mosaicism (Acuna-Hidalgo et al., 2016; Rahbari et al., 2016). Thus, great importance should be attached to evaluate its recurrent risk for implementing prevention.

The pathogenic variant allele fractions in male patients with paternal germline mosaicism have been reported from very low- to moderate-level (0.03%–39%) (Yang et al., 2017). Considering the low-level mosaicism (<3%) and the high-frequency recurrence of the paternal pathogenic variant, we suspect that sperm with this variant may possess an advantage in competing with normal sperm during fertilization. Moreover, with the low level of mosaicism in sperm and high frequency of carriage of variants in offspring and embryos, the possibility of false positive results from linkage analysis or allele drop-out (ADO) from Sanger’s sequencing could not be excluded. In addition, the inconsistent results from haplotype linkage analysis (Figure 2A) and Sanger’s sequencing (Figure 2C) could blame the same reason.

Breuss et al. recently proposed three types of sperm mosaicism based on the timing of originally arising in sperm lineage (Breuss et al., 2021). Type I sperm mosaicism appears during sperm terminal differentiation and never recurs. Type II sperm mosaicism develops in proliferating spermatogonial stem cells (SSCs) and includes extant clonally (IIa) or those under positive selection (IIb), similar to the selfish sperm hypothesis. Multiple inheritance is uncommon for IIa, but the selection advantages of IIb may lead to SSC clone over-proliferation and the possibility of population-wide recurrence. However, type III, which occurs following or prior to primordial germ cell, can lead to intrafamilial recurrence. Furthermore, type III could be divided into IIIa and IIIb; the former shows the mosaicism detected in sperm and somatic cells, whereas the latter shows the mosaicism restricted to sperm. With the readily recurring DNMs across siblings and the low-level mosaicism limited to sperm, we speculate that the DNMs in this family originated from the type IIb sperm mosaicism or earlier stage in the father. In addition, since we only collected peripheral blood and saliva samples of the father, the possibility that he has IIIa type sperm mosaicism (somatic and sperm mosaicism) could not be excluded as many other tissues such as hair or nail are not involved.

The current study could not fully explain the high frequency of recurrence for such low-level mosaicism in the sperm. Further studies are warranted to clarify the potential mechanisms.

In conclusion, this is the first reported pregnancy with a genetically normal child achieved *via* PGT for a germline mosaicism father. It was completed by the collaboration of multidisciplinary experts, including fertility doctors, geneticists, obstetricians, and prenatal diagnosticians in our hospital. Our case with this healthy child opens the possibility of preventing the recurrent monogenic disease caused by gonadal mosaicism. Moreover, couples who have given birth to a child with DNMs may need genetic counselling before having another child to avoid or reduce the risk of recurrence. In particular, the possibility of gonadal mosaicism should be considered for the recurrence of DNMs.

## Data Availability

The original contributions presented in the study are included in the article; further inquiries can be directed to the corresponding authors.
